# Membrane-Based Inverse Transition Cycling: An Improved Means for Purifying Plant-Derived Recombinant Protein-Elastin-Like Polypeptide Fusions

**DOI:** 10.3390/ijms12052808

**Published:** 2011-04-29

**Authors:** Hoang Trong Phan, Udo Conrad

**Affiliations:** Leibniz Institute of Plant Genetics and Crop Plant Research (IPK), Corrensstrasse 3, Gatersleben 06466, Germany; E-Mail: hoang@ipk-gatersleben.de

**Keywords:** transgenic plants, avian influenza, H5N1, nanobody, ELPylation, Inverse Transition Cycling

## Abstract

Elastin-like peptide (ELP) was fused to two different avian flu H5N1 antigens and expressed in transgenic tobacco plants. The presence of the ELP tag enhanced the accumulation of the heterologous proteins in the tobacco leaves. An effective membrane-based Inverse Transition Cycling was developed to recover the ELPylated antigens and antibodies from plant material. The functionality of both the ELPylated neuraminidase and an ELPylated nanobody was demonstrated.

## 1. Introduction

The use of transgenic plants to synthesize antigens and antibodies was first reported over 20 years ago [1,2], and since this time, various methods have been developed to generate both plant-based vaccines and plantibodies (for review see [3–7]). Using plants to make these molecules, offers significant advantages over mammalian cell-based systems in scale, economy and product safety, as well as providing a simple means of storage and distribution in the form of the seed (for review see [4,8]). Target extraction, recovery and purification, which normally involves chromatography and its associated labor and capital equipment, represents over 80% of the overall production cost [9]. These costs are independent of the primary production system, but *in planta* systems are less demanding than *in vitro* ones with respect to the upstream components, as they avoid the need for fermentation equipment and culture media [8]. As a result, the per gram cost of therapeutic proteins produced by mammalian cells is substantially higher than that from plants [10]. The major technical bottleneck associated with *in planta* production lies in the down-stream processing procedure [11]. Here the major challenges concern firstly the removal of contaminating proteins (especially proteases), carbohydrates, oils, phenolic compounds, phytic acid, nucleic acid and other trace products, and secondly the low concentration of the target in the initial aqueous extraction [12]. Thus, successful purification depends heavily on inducing a high concentration of the target molecule in the plant material.

The fusion of transgenic proteins to elastin-like peptides (ELPs) is known to enhance the accumulation of transgenic proteins *in planta* (for review see [13,14]), and ELPylated proteins are known to induce immune responses [15]. ELPylation has the further benefit of simplifying the subsequent purification *via* Inverse Transition Cycling (ITC), a method based on the inherent reversibility of ELPylation (for review see [13]). This approach has been successfully demonstrated for the expression and purification of vaccines [15], complete immunoglobulins [16,17], antibody fragments [18,19] and several other proteins (for review see [13,14]). The centrifugation-based ITC method (cITC) relies on the precipitation of ELPylated proteins by a combination of salting, heating, centrifugation and resolubilization in the absence of salt at a lower temperature [20], a method which has been further improved by the use of microfiltration to isolate the precipitate [21]. Recently, we have shown that an *in planta* produced ELPylated cameloid-based nanobody against hTNFα can be purified by cITC and size exclusion chromatography, and that this molecule was able to neutralize the cytotoxic effect of hTNFα in human cells and prevent sepsis in a mouse model. The ELPylated nanobody showed a significant enhancement of half-life [19].

Avian flu has grown in importance as a disease affecting both domestic animals and humans (for review see [22,23]). A full-length hemagglutinin which induces the production of virus-neutralizing antibodies in mice has been produced *in planta* [24,25], and the same hemagglutinin has been shown to protect ferrets against the virus [26,27]. Similarly, plant-produced hemagglutinin vaccines are effective in chickens in a challenge study [28]. Plant-produced transgenic antigens have been purified from the plant matrix using either Protein A based affinity chromatography *via* Fc fusions [29], Ni-column chromatography and anion exchange chromatography [27], or a combination of two phase separations, several membrane filtration steps and gel filtration [28]. Here we demonstrate the purification of both ELPylated avian flu hemagglutinin and neuraminidase (NA) synthesized in transgenic tobacco plants using a membrane-based ITC (mITC) method.

## 2. Results and Discussion

### 2.1. Generation of Transgenic Tobacco Plants Expressing Hemagglutinin and NA Sequences

**Figure 1.**
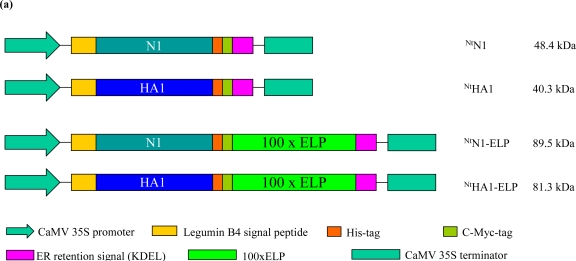

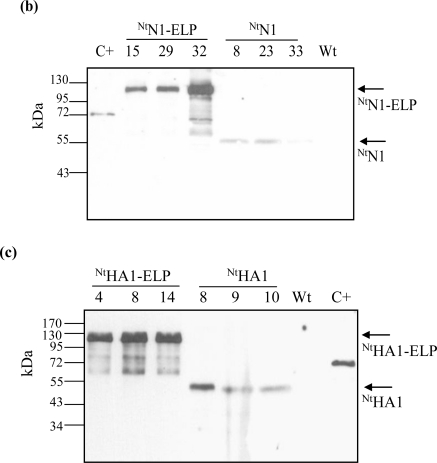
The heterologous expression of ^Nt^N1 and ^Nt^HA1 in tobacco. (**a**) Schematic representation of the expression cassettes. The N1 and HA1 subunit encoding sequences were cloned in frame with a legumin signal peptide (SP), a his tag, a c-myc-tag, the ER retention signal (KDEL) and either with or without 100 repeats of the ELP pentapeptide VPGXG. N1: NA; HA1: HA1 subunit 1 of hemagglutinin subtype 5; (**b**) ^Nt^N1-ELP and ^Nt^N1 protein, as detected by Western blotting using, respectively, 2.5 and 25 μg total soluble protein (TSP); (**c**) ^Nt^HA1-ELP and ^Nt^HA1, as detected by Western blotting using, respectively, 2.0 and 30 μg TSP. The numbers refer to independent primary transgenic plants. Wt: wild type tobacco; C+: 1ng ^Nt^anti-hTNFα-VHH-ELP used as a Western blot standard. Arrows indicate the target recombinant proteins.

The avian flu antigens neuraminidase and hemagglutinin are important targets for the production of neutralizing antibodies [24,30,31], (for review see [32]). Expression cassettes were designed to synthesize hemagglutinin H5 (HA1 (A/Hatay/2004/(H5N1), GenBank Q5QQ29) and NA N1 ((A/Hatay/2004/(HHN1), GenBank Q5QQ28) *in planta*. The transgenic protein was targeted to the endoplasmic reticulum by ER retention, as this ensures high and stable accumulation [33], (for review see [34]). ELPylated versions of both antigens were also included to both enhance the level of *in planta* expression and to permit purification *via* ITC (Figure 1a, for review see [13]). Transgenic plants were produced with all four constructs using *Agrobacterium*-mediated transformation (Table 1). Whereas the *in planta* expression of hemagglutinin (H5) has already been achieved elsewhere [24,28,29], we believe that this is the first report of *in planta* synthesis of NA N1.

ELPylation enhanced the expression of both NA and hemagglutinin, as it has been documented to achieve for a range of other transgenic proteins including antigens and antibodies [16–19,35,36]. The extent of the enhancement appears to depend on the number of ELP repeats included in the expression cassette [36], and here, the use of 100 repeats produced a high level of expression of both antigens (Table 1, Figure 1).

### 2.2. Purification of ^Nt^HA1-ELP, ^Nt^N1-Elp Using Centrifugation-Based ITC (cITC)

**Figure 2.**
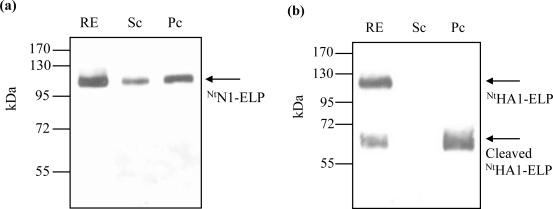
cITC purification, as demonstrated by Western blot analysis. (**a**) ^Nt^N1-ELP; (**b**) ^Nt^HA1-ELP. RE: raw extract; Sc: Supernatant after heat centrifugation; Pc: Proteins captured in the pellet after heat centrifugation and solubilization in water. Arrows indicate the target recombinant proteins.

Both antigens were readily purified by cITC. The NA antigen was accompanied by other plant proteins (Figures 2a, 4a), resulting in a purification efficiency of 40.0% (in terms of protein amount) and 33.7% (in terms of enzymatic activity) (Table 2). For the hemagglutinin antigen, the method resulted in the near complete disappearance of the full-length hemagglutinin-ELP fusion; what remained was one cleavage product consisting of the ELP repeats and the cmyc-tag (Figure 2b). The recovery efficiency of this protein was high (Table 2). In general, the recovery efficiency of cITC was in accordance with the literature [19,36].

### 2.3. Purification Using Membrane-Based ITC (mITC)

**Figure 3.**
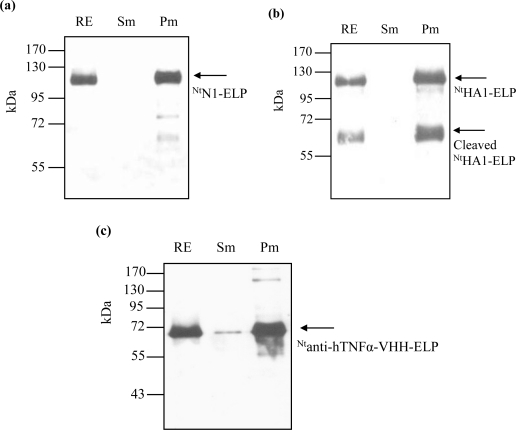
mITC purification, as demonstrated by Western blot analysis. (**a**) ^Nt^N1-ELP; (**b**) ^Nt^HA1-ELP; (**c**) ^Nt^anti-hTNFα-VHH-ELP. RE: Raw extract; Sm: Supernatant raw extract passed through a 0.2 μm cellulose acetate membrane at room temperature; Pm: Proteins eluted from the membrane by ice-cold water. Arrows indicate the target recombinant proteins.

**Figure 4.**
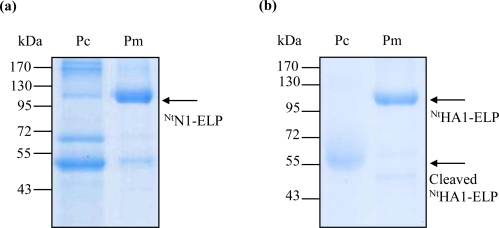
In gel staining (Coomassie Blue) analysis of purification. (**a**) ^Nt^N1-ELP; (**b**) ^Nt^HA1-ELP. Pm: proteins eluted from the membrane by water; Pc: proteins captured in the pellet after heat centrifugation and solubilization in water. Arrows indicate the target recombinant proteins.

**Figure 5.**
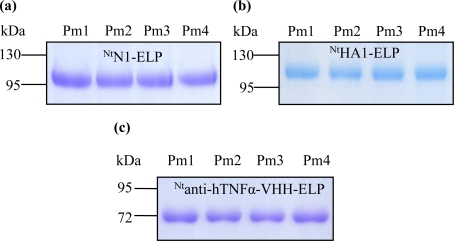
Robustness of the mITC purification. (**a**) ^Nt^N1-ELP; (**b**) ^Nt^HA1-ELP; (**c**) ^Nt^anti-hTNFα-VHH-ELP. Pm1-Pm4 refer to the four replicated purifications.

To overcome the proteolytic cleavage and/or partial denaturation of the ELPylated protein which affected the cITC purification (probably caused by a higher than optimal temperature regime), we adapted the filter-based ITC method described by Ge *et al.* [21]. For each of the three transgenic products (^Nt^HA1-ELP, ^Nt^N1-ELP and ^Nt^anti-hTNFα-VHH-ELP), the expected size molecule was successfully purified (Figure 3). Losses due to filtration at room temperature and the presence of 2 M NaCl were either insignificant (Figure 3a,b) or, at worst, minimal (Figure 3c). Since the re-solubilization rate was very high, the mITC purification efficiency was excellent (Table 2). For NA in particular, the procedure led to a large improvement in the degree of purity achieved (Figure 4a). For hemagglutinin, some proteolytic cleavage still occurred, but most of the product remained intact (Figure 4b). The robustness of the mITC method was proven by repeating the procedure four times for each target (Figure 5).

**Figure 6.**
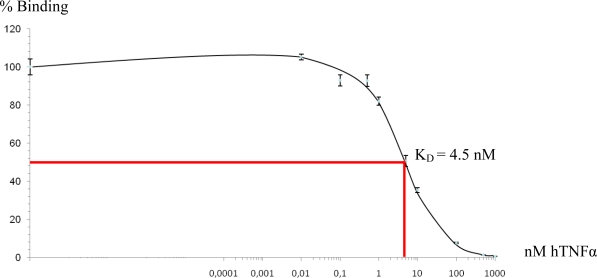
The binding behavior of ^Nt^anti-hTNFα-VHH-ELP to human TNFα, as assessed by competitive ELISA. ^Nt^anti-hTNFα-VHH-ELP was mixed with various concentrations of hTNFα. The solid phase binding of c-myc tagged ^Nt^anti-hTNFα-VHH-ELP to hTNFα was quantified. Binding without competition from free hTNFα was set to 100%. Standard deviations shown as bars.

Purification procedures need to be optimized not only with respect to their recovery efficiency, but also to maintain biological activity. The three ELPylated proteins expressed *in planta* and purified by mITC were highly soluble even at relatively high concentrations. The enzymatic activity of the NA, assessed on the basis of its ability to cleave 2′-(4-methylumbelliferyl)-α-d-*N*-acetylneuraminic acid, was over four fold that of the cITC purified equivalent (Table 3). The binding behavior of the mITC purified nanobody ^Nt^anti-hTNFα-VHH-ELP was investigated by a competitive ELISA, in which it exhibited a dissociation constant of ∼4.5 nM (Figure 6), comparable to the behavior of its cITC purified equivalent (4 nM, [19]).

We have demonstrated here that a refinement of the mITC method permits a rapid, inexpensive and efficient means of purifying *in planta* expressed ELPylated transgenic proteins. The purified products showed a high degree of integrity, were highly soluble and exhibited excellent biological activity in terms of their binding or their enzymatic cleavage. Further developments will concentrate on a scaling up of the procedure to verify that this technology is able to circumvent the major bottleneck affecting the *in planta* production of antigens and antibodies [11].

## 3. Experimental Section

### 3.1. Plant Transformation Vectors

The hemagglutinin (HA) sequence encoding amino acids 2–568 of the A/Hatay/2004/(H5N1) strain of the avian flu virus was optimized for expression in tobacco plants and synthesized by GENEART AG (Regensburg, Germany). This sequence was designated pPCRScript-opHA0. Truncated hemagglutinin HA1 and NA N1 sequences were obtained by a two-step cloning strategy. First, a polyhistidine affinity purification tag (6xHis) was added to the *C*-terminus of TBAG to create the vectors pRTRA-TBAG and pRTRA-TBAG-ELP [15] comprising the constitutively expressing cauliflower mosaic virus (CaMV) 35S promoter, the legumin B4 (LeB4) signal peptide (SP), TBAG, a c-myc tag, respectively 0 and 100 copies of ELP, and the KDEL ER retention signal. Next, the DNA sequence encoding A/Hatay/2004/(H5N1) HA1 (amino acids 17–342) and NA1 (amino acids 38–449) were PCR amplified using, respectively pPCRscript-opHA0 and pPCR-N1 (GenBank accession AJ867075) as template; the amplicon was inserted into pRTRA-TBAG and pRTRA-TBAG-ELP to replace the sequence DNA encoding TBAG to form the four expression cassettes pRTRA-35S-HA1, pRTRA-35S-HA1-ELP, pRTRA-35S-N1 and pRTRA-35S-N1-ELP (Figure 1a). Each cassette was then *Hin*dIII digested and inserted into the pCB301-based shuttle vector pCB301-Kan [16,37].

### 3.2. Plant Transformation

The pCB301-Kan vectors were electroporated into *Agrobacterium tumefaciens* strain C58C1 (pGV2260)[38] which was then used to transform the tobacco cultivar SNN *via* a leaf disk method [39]. Transgenic plants were cultured on Murashige and Skoog medium containing 50 mg/L kanamycin and analyzed by Western blot using a monoclonal antibody anti-c-myc antibody. Transgenic high-producer tobacco lines were selected and further propagated.

### 3.3. ITC Purification of ELP Fusion Proteins

Frozen leaf (150 g) was ground with mortar and pestle in liquid nitrogen and homogenized in 220 mL ice-cold 50 mM Tris-HCl (pH 8.0). A Complete Protease Inhibitor Cocktail tablet (Roche, Germany) was added to the slurry, which was then cleared by centrifugation (75,600 g, 30 min, 4 °C) before the addition of NaCl to a final concentration of 2 M. The cold extract with 2 M NaCl was centrifuged again at 75,600 g for 30 min at 4 °C. The solution was then passed through a 0.22 μm polyethersulfone membrane (Corning, USA) with the temperature maintained at 4 °C, to produce a pre-treated extract. For the cITC, the pre-treated extract was first incubated for 30 min at 40 °C (for ^Nt^N1-ELP purification) or 55 °C (^Nt^HA1-ELP) to induce aggregation of the antigen-ELP fusions, which were then precipitated by centrifugation (8000 g, 30 min, 40 °C or 55 °C, respectively). The resulting pellet was dissolved in water at 4 °C, and any insoluble matter removed by centrifugation (15,000 g, 30 min, 4 °C). For the mITC, the pre-treated extract was warmed to room temperature, and passed through a 0.2 μm cellulose acetate membrane (Sartorius Stedim, Goettingen, Germany) using a vacuum pump (Vacuubrand, Germany). The membrane was washed twice with 2 M NaCl to remove contaminating proteins. Ice-cold Millipore-Q water was then passed through the filter to elude the protein-ELP fusions.

### 3.4. SDS-PAGE and Western Blotting

Frozen leaf discs were homogenized in a Mixer Mill MM 300 (Retsch, Haan, Germany) and the resulting powder suspended in SDS sample buffer (72 mM Tris, 10% v/v glycerine, 3% w/v SDS, 5% v/v β-mercapethanol, 0.25 μM bromophenol blue, pH 6.8), held at 95 °C for 10 min and then centrifuged (19,000 g, 30 min, 4 °C). The concentration of total soluble protein (TSP) present was determined using the Bradford assay (Bio-Rad, Munich, Germany). Extracted plant proteins were separated by reducing SDS-PAGE (10% polyacrylamide) and electrotransferred to nitrocellulose membranes. After blocking with 5% w/v fat-free milk powder dissolved in TBS (20 mM Tris, 180 mM NaCl, pH 7.8), the membranes were incubated for 2 h at room temperature with a monoclonal anti-c-myc antibody. The presence of this antibody was detected by the addition of a 1:2000 dilution of HRP-conjugated sheep anti-mouse IgG (Amersham, Germany), and the signal visualized using the ECL method (Amersham Biosciences). Each membrane was washed three times between each step with TBS containing 0.5% w/v fat-free milk, except for the penultimate (TBS only) and final (phosphate-buffered saline, PBS) washes. Antibodies were diluted in TBS, 5% w/v fat-free milk powder.

### 3.5. Determination of Purification Efficiency

The purification efficiency of the various recombinant protein-ELP fusions was assessed by sampling the plant extract both before and after the cellulose acetate filtration (mITC method), or before and after the hot centrifugation (cITC method). The samples were serially diluted to allow a semi-quantitative Western blotting-based analysis (as described in 3.4). Band intensities were compared by image densitometry, using totalLab TL100 software (Nonlinear Dynamics, USA).

### 3.6. ^Nt^N1-ELP Activity Assay

A fluorescence-based assay was used to measure NA activity, as described elsewhere [40,41]. The assay was formed by adding a 10 μL aliquot of the ^Nt^N1-ELP sample to an equal volume of 32.5 mM MES, 4 mM CaCl2, pH 6.5. The enzymatic reaction was then initiated by the addition of 30 μL 2′-(4-methylumbelliferyl)-α-d-*N*-acetylneuraminic acid (Sigma, St. Louis, MO, USA). After incubation at 37 °C for 30 min, the reaction was stopped by the addition of 150 μL 100 μM glycine (pH 10.7) in 25% ethanol. The release of 4-methylumbelliferone (4-MU) was measured spectrophotometrically at 355 and 460 nm, and quantification was based on a standard curve prepared with pure free 4-MU (Sigma, St. Louis, MO, USA). One unit of NA was defined as the quantity able to release 1 μM 4-MU per min at 37 °C. To determine the efficiency of ^Nt^N1-ELP purification based on its NA activity, total protein was precipitated from the initial plant extract with ammonium sulfate, and pelleted by centrifugation (38,000 g, 30 min, 4 °C). The pellet was re-solubilized in PBS, and the sample treated as above to determine NA activity. The % recovery of ^Nt^N1-ELP based on NA activity was defined as the ratio between the NA activity before and after purification.

### 3.7. Competitive ELISA

Microtitre plates (ImmunoPlate Maxisorp, Nalge Nunc International, Roskilde, Denmark) were coated with 1 μg/mL recombinant human TNFα in PBS and incubated overnight at room temperature. After blocking with 3% w/v bovine serum albumin (BSA), 0.05% v/v Tween20 in PBS (PBST) for 2 h, pre-determined quantities of ^Nt^anti-hTNFα-VHH-ELP mixed with various concentrations of hTNFα in 1% w/v BSA in PBST were added to each well, and the plates incubated for 1.5 h at 25 °C. Bound ^Nt^anti-hTNFα-VHH-ELP was visualized by treatment with anti-c-myc antibody and rabbit anti-mouse IgG alkaline phosphatase conjugate (SIGMA-Aldrich, St. Louis, MO, USA) diluted in 1% w/v BSA in PBST. The enzymatic substrate was pNP phosphate, and the absorbance was measured after a 60 min incubation at 37 °C. Five replicates were measured for each concentration of free hTNFα used for competition.

## 4. Conclusions

Avian flu virus H5N1 hemagglutinin (H5) and neuraminidase (NA, N1) have been expressed transgenically in tobacco. The level of heterologous expression was greatly enhanced by fusing 100 ELP repeats to the C-terminus of each protein. The enhanced expression of the two main targets for neutralizing antibodies of the Avian flu virus in transgenic tobacco allows the development of combined vaccination strategies with both antigens.

Refinements to the mITC method produced an efficient, rapid and non-expensive means of purifying the *in planta* synthesized ELPylated proteins. The critical improvements to the purification protocol were the clearance of the initial extract, a second clearance step after addition of NaCl to 2 M in the cold, a rapid and efficient precipitation reaction, and the elution of the target from the membrane with ice-cold water. The integrity of the resulting products was high, and they also showed a good level of biological activity. Immunization experiments will be required to show that *in planta* produced ELPylated hemagglutinin and neuraminidase can provide effective protection against infection.

The recovery efficiency of the membrane-based ITC procedure was very high in all three examples. This is essentially true for the purification of ^Nt^anti-hTNFα-VHH-ELP. Active nanobody with comparable affinities has been produced by centrifugation-based ITC, too, but with a ten times lower recovery efficiency (Table 2). The mITC method described in this paper could also be adapted in future to the purification of ELPylated complete antibodies, suitable for therapeutic purposed [16,17].

In general we conclude that ELPylation is an appropriate strategy for the development of plant-based vaccines and plantibodies. Scaling up experiments will now be necessary to confirm that this purification approach will be able to circumvent the major bottleneck which at present inhibits the *in planta* production of antigens and antibodies, *i.e.*, the down-stream costs [11].

## Figures and Tables

**Table 1.** Transgenic tobacco plants expressing neuraminidase N1, hemagglutinin (H5, HA1) or the respective ELPylated fusion proteins.

**Transgene**	**Number of Regenerated Plants**	**Number of Transgene Expressing Plants**	**TransgeneExpression (%TSP)**
^Nt^N1	42	23	0.004%
^Nt^N1-ELP	37	23	0.2%
^Nt^HA1	36	22	0.01%
^Nt^HA1-ELP	88	67	0.3%

Transgenic proteins were detected by Western blot.

**Table 2.** Efficiency of purification of various recombinant target proteins by mITC and cITC.

**Recombinant proteins**	**Recovery (%)**	
	**mITC**	**cITC**
^Nt^HA1-ELP	95*	98*
^Nt^N1-ELP	92.5**–94*	33.7**–40*
^Nt^anti-hTNFα-VHH-ELP	98*	11* [19]

Determined by

* amount (Western blot) or

** activity.

**Table 3.** Enzymatic activity of ^Nt^N1-ELP.

**Status of ^Nt^N1-ELP**	**Total protein (mg)**	**Total activity (U)**	**Recovery rate (%)**	**Specific activity (U mg^−1^)**
Raw extract	3521.9	736.5	100	0.21
Purified ^Nt^N1-ELP by mITC	20.3	681.1	92.5	33.6
Purified ^Nt^N1-ELP by cITC	31.2	248.5	33.7	7.96

The ^Nt^N1-ELP activity was determined by fluorescence assay before and after purification by mITC or cITC. Data were calculated for 1 kg of leaf material.
